# Elucidating the nociceptive role of CGRP in migraine headache

**DOI:** 10.1093/brain/awag008

**Published:** 2026-01-08

**Authors:** Agustin Melo-Carrillo, Andrew M Strassman, Rami Burstein

**Affiliations:** Department of Anesthesia, Critical Care and Pain Medicine, Beth Israel Deaconess Medical Center, Boston, MA 02115, USA; Department of Anesthesia, Harvard Medical School, Boston, MA 02115, USA; Department of Anesthesia, Critical Care and Pain Medicine, Beth Israel Deaconess Medical Center, Boston, MA 02115, USA; Department of Anesthesia, Harvard Medical School, Boston, MA 02115, USA; Department of Anesthesia, Critical Care and Pain Medicine, Beth Israel Deaconess Medical Center, Boston, MA 02115, USA; Department of Anesthesia, Harvard Medical School, Boston, MA 02115, USA

**Keywords:** trigeminal ganglion, nociceptors, pain, meninges, central sensitization

## Abstract

Calcitonin gene-related peptide (CGRP) is thought to be a key player in the pathogenesis of migraine, but there is a fundamental mystery in that the known neuronal actions of CGRP do not account for how it causes pain. We now report the first finding of CGRP-induced nociceptive neuronal activation using a novel method of intra-carotid infusion to achieve a more targeted delivery to cranial tissues.

Single-unit recordings were performed in anaesthetized rats to measure CGRP effects on first- and second-order trigeminovascular neurons. CGRP was administered via intra-carotid infusion. Neuronal activation and sensitization were assessed by spontaneous firing rates and responses to mechanical stimulation of dural and facial receptive fields. Lidocaine was applied locally to the dura or trigeminal ganglion at varying time points to determine the peripheral contribution to CGRP-induced activity.

Intra-carotid CGRP infusion (5 µg/kg/min, 20 min) activated 62% of Aδ-fibres and 56% of C-fibres, with significant increases in firing rates beginning within the first 30 min for Aδ-fibres and after 1 h for C-fibres. It also activated 75% of central trigeminovascular neurons, significantly increasing spontaneous firing and sensitizing dural and facial receptive fields. Similar effects were produced by CGRP injection into the trigeminal ganglion. These effects of CGRP were impeded by local anaesthetic blockade of the dura or trigeminal ganglion before but not 1 h after CGRP infusion. No significant sex differences were found in baseline firing or in the magnitude and timing of CGRP-induced responses across all neuron types.

These findings provide the first evidence of peripheral nociceptive neuronal activation by CGRP, with a site of action in the meninges, and support a rationale for early, peripherally acting CGRP-targeted migraine treatments.

## Teaser

Melo-Carrillo *et al.* show that CGRP directly activates peripheral nociceptors in the meninges, triggering migraine pain. The findings provide a rationale for using early, peripherally acting CGRP-blocking treatments to stop migraine.

## Introduction

Calcitonin gene-related peptide (CGRP) is the most abundant of the neuropeptides expressed in primary afferent sensory neurons of the dorsal root and trigeminal ganglia.^1-3^ CGRP and its receptors are present in peripheral tissues throughout the body, as well as in regions of the CNS.^2,4-6^ In general, CGRP has not been a major focus of research into treatments for pain, with the one outstanding exception of migraine. CGRP is arguably the most important single molecule in current research into migraine mechanisms; it is a strong vasodilator^7,8,8^ capable of provoking migraine-like headache in patients.^9,10^ Consequently, CGRP and its receptors have been targeted in the most recent generation of migraine therapeutic agents.^11-19^ Paradoxically, CGRP has not been found to be effective in producing excitation of peripheral nociceptive neurons, including the sensory neurons that innervate the meninges,^20^ a sensory innervation that has been implicated in the generation of migraine headache.^8,21,22^ The current study addresses this fundamental inconsistency between clinical and preclinical data by testing the effects of CGRP on first- and second-order nociceptive neurons of the meningeal sensory pathway using a novel method of infusion via the intra-carotid artery in order to achieve a more targeted delivery to the cranial tissues.

## Materials and methods

Experiments were approved by the Beth Israel Deaconess Medical Center and Harvard Medical School standing committees on animal care and were in accordance with the National Institutes of Health’s Guide for the care and use of laboratory animals.

### Surgical preparation

Male and female Sprague Dawley rats (250–320 g, 10 to 12 weeks old) were anaesthetized with urethane (0.9–1.2 g/kg, intraperitoneal). They were fitted with an intratracheal tube to allow artificial ventilation (0.1 l/min of O_2_). Two cannulas were surgically implanted in the animals for later infusion of drugs: an intrafemoral vein cannula and an intra-carotid artery cannula (common carotid artery). Rats were placed in a stereotaxic apparatus, and core temperature was kept at 37°C using a heating blanket. End-tidal CO_2_ was monitored continuously and kept within physiological range (3.5–4.5 pCO_2_). Once stabilized, rats were paralysed with rocuronium bromide (10 mg/ml, 2.1 ml/h continuous intravenous infusion) and ventilated. In all experiments, for stimulation of the cranial dura later in the experiment, a 5 × 5 mm opening was carefully carved in the parietal and occipital bones in front and behind the lamboid suture directly above the left transverse sinus. The exposed dura was kept moist using a modified synthetic interstitial fluid containing the following (in mM): 135 NaCl, 5 KCl, 1 MgCl_2_, 5 CaCl_2_, 10 glucose and 10 HEPES, pH 7.2.

### Peripheral recordings

In experiments in which recordings were made from peripheral neurons in the left trigeminal ganglion, a craniotomy was made on the right (contralateral) side to allow the microelectrode to be advanced through the contralateral cortex to reach the ganglion, using an angled approach. This was done to avoid damage to the ipsilateral cortex. The craniotomy was made to allow electrode insertions into the right cortex covering an area of 1–3 mm caudal to bregma and 1.5–3 mm lateral. The dura covering this area of cortex was removed to allow microelectrode insertion.

### Central recordings

In experiments in which recordings were made from central neurons in the left C1–C2 dorsal horn (spinal trigeminal nucleus), a segment of the spinal cord between the obex and C2 was uncovered from overlying tissues, stripped of the dura mater and kept moist with synthetic interstitial fluid.

Experiments were only performed in cases in which the physiological condition of the rats (heart rate, blood pressure, respiration, end-tidal CO_2_) and the neuronal isolation signal (signal-to-noise ratio ∼ 1:3) were stable throughout the experimental period. At the conclusion of all central experiments, a small lesion was created at the recording site [anodal DC (direct current) of 15 μA for 15 s], and its localization in the dorsal horn was determined post-mortem using histologic analysis. Only one neuron was studied in each animal.

### Identification and characterization of peripheral and central trigeminovascular neurons

#### Peripheral recordings

A platinum-coated tungsten microelectrode (impedance 150–300 kΩ) was advanced into the trigeminal ganglion for single-unit recording. To reach the ganglion via a contralateral approach, the electrode was angled medially 21° and was advanced through the contralateral cortex (see earlier for coordinates). Dural afferent neurons in the ganglion were identified by their constant latency response to single-shock stimulation delivered to the dura overlying the ipsilateral transverse sinus with a bipolar stimulating electrode (0.5 ms pulse, 5 mA, 0.75 Hz). The response latency was used to calculate conduction velocity, based on a conduction distance to the trigeminal ganglion of 12 mm. Neurons were classified as either Aδ-fibres (conduction velocity 1.5–5 m/s) or C-fibres (conduction velocity < 1.5 m/s). Neurons with conduction velocity > 5 m/s were not studied. Spike 2 software (Cambridge Electronic Design) was used for acquisition and waveform discrimination of the electrically evoked spikes, and for offline analysis. Mechanical receptive fields of dural afferents were identified by probing the dura with a von Frey hair (4.19 g). For the non-dural neurons, receptive fields were identified by brushing the skin. Only neurons for which a mechanical receptive field could be identified were selected for study.

#### Central recordings

To record neuronal activity, a tungsten microelectrode (impedance 1–4 mΩ, FHC Inc.) was lowered repeatedly into the spinal trigeminal nucleus (C1–C2 dorsal horn) in search of central trigeminovascular neurons receiving input from the dura. Trigeminovascular neurons were first identified based on their responses to electrical stimulation of the dura. They were selected for the study if they exhibited discrete firing bouts in response to ipsilateral electrical (0.1–3.0 mA, 0.5 ms, 0.5 Hz pulses) and mechanical (with a calibrated von Frey monofilament) stimulation of the exposed cranial dura.

Dural receptive fields were mapped by indenting the dura (4.19 g von Frey hair monofilament). Points at which dural indentation produced a response in >50% of the trials were considered inside the neurons’ receptive field. Cutaneous receptive fields were mapped by applying innocuous and noxious mechanical stimulation to all facial skin areas as described previously.^8^ An area was considered outside the receptive field if no stimulus produced a response in >50% of the trials. Responses to mechanical stimulation of the skin were determined by applying brief (10 s) innocuous and noxious stimuli to the most sensitive portion of the cutaneous receptive field. Innocuous stimuli consisted of slowly passing a soft bristled brush across the cutaneous receptive field (one 5-s brush stroke from caudal to rostral and one 5-s brush stroke from rostral to caudal) and pressure applied with a loose arterial clip. Noxious stimuli consisted of a pinch with a strong arterial clip. More intense or prolonged stimuli were not used to avoid inducing prolonged changes in spontaneous neuronal discharge or response properties. Two classes of neurons were thus identified: wide dynamic range (WDR) neurons (incrementally responsive to brush, pressure and pinch); and high-threshold (HT) neurons (unresponsive to brush). A real-time waveform discriminator was used to create and store a template for the action potential evoked in the neuron under study by electrical pulses on the dura; spikes of activity matching the template waveform were acquired and analysed online and offline using Spike 2 software (Cambridge Electronic Design, Cambridge, UK).

### CGRP infusion protocol

CGRP (α calcitonin gene-related peptide rat; Sigma Aldrich) was infused using the intra-carotid artery cannula at a rate of 50 ng/kg/min for 20 min (dose from human studies)^8^ with a programmable pump (World Precision Instruments). To avoid any blood clots while the cannula was inserted and was not infusing CGRP/vehicle, the cannula was filled with sterile catheter lock solution (heparin 500 units/ml in 50% dextrose).

### Trigeminal ganglion microinjections

Microinjections were made into the trigeminal ganglion (ipsilateral to the central recordings), using a UMP-3 microinjector and Micro 4 controller (World Precision Instruments). The injected solution (lidocaine/bupivacaine or CGRP mixed with Chicago Blue 1%, 10 μl) completely infiltrates the space surrounding the ganglion, which is enclosed within a continuous connective tissue sleeve (dura and epineurium). A photograph was made post-mortem to document the degree of filling after each experiment.

### Data analysis

Neuronal activity was analysed to measure amplitude, latency and duration of responses to CGRP infusion. To determine neuronal responses to CGRP, the mean firing frequency occurring before the CGRP infusion (calculated from measuring the spontaneous activity 30 min) was compared with the mean firing frequency recorded in 30 min intervals after the CGRP infusion. A neuron was considered activated if its mean firing rate after CGRP exceeded its mean baseline activity by 1 standard deviation for a period >10 min, which translated to approximately >33% increase in activity. Mean firing rates of respective values were compared using non-parametric repeated-measures test (Friedman test) and *post hoc* analysis (Tukey's honestly significant difference). The level of significance was set at 0.05.

## Results

### Activation and sensitization

Different routes of CGRP infusion were tested for its effects on the firing activity of first- and second-order neurons of the meningeal sensory pathway, in the trigeminal ganglion and upper cervical dorsal horn, respectively, in single-unit recordings in anaesthetized rats. When administered either by intraperitoneal injection (100 µg/kg), or by intravenous infusion at the low dose used in human headache provocation studies (5 µg/kg/min for 20 min), CGRP failed to produce activation of first- or second-order neurons [0/12 (7 females, 5 males) and 0/11 (7 females, 4 males), respectively, intraperitoneal injection; 0/10 (6 females, 4 males) and 0/10 (6 females, 4 males), respectively, intravenous infusion], consistent with previous studies.^20^

**Figure 1:**
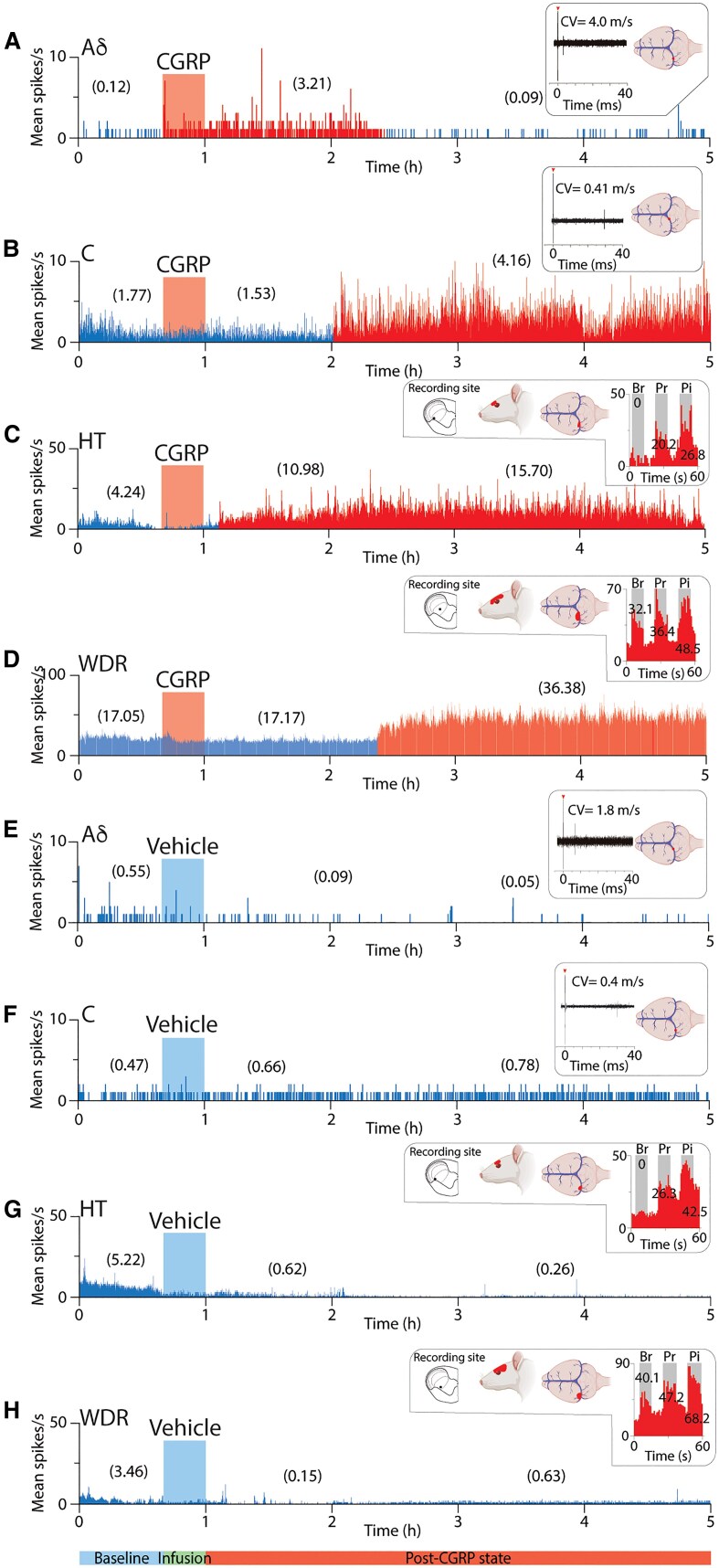
**Intra-carotid CGRP infusion activates peripheral and central nociceptive neurons with dural receptive fields**. Plots of firing rate in examples of individual Aδ, C, HT and WDR neurons tested for activation by intra-carotid infusion of CGRP (50 ng/kg/min for 20 min) (**A**–**D**) or saline (**E**–**H**). Activity plotted in red indicates significant increase above baseline (1 standard deviation). Note that the increase in activity occurs earlier in Ad and HT neurons as compared with C-fibres and WDR neurons. *Insets* for Aδ- and C-fibres (**A**, **B**, **E** and **F**) show dural receptive fields, and spike evoked by dural shock. *Insets* for HT and WDR neurons (**C**, **D**, **G** and **H**) show dural and facial receptive fields, dorsal horn recording site and firing in response to facial stimulation. CGRP = calcitonin gene-related peptide; CV = conduction velocity; HT = high threshold; WDR = wide dynamic range. Figure partially created in BioRender. Melo Carrillo, A. (2026) https://BioRender.com/20s2oqb.

**Figure 2:**
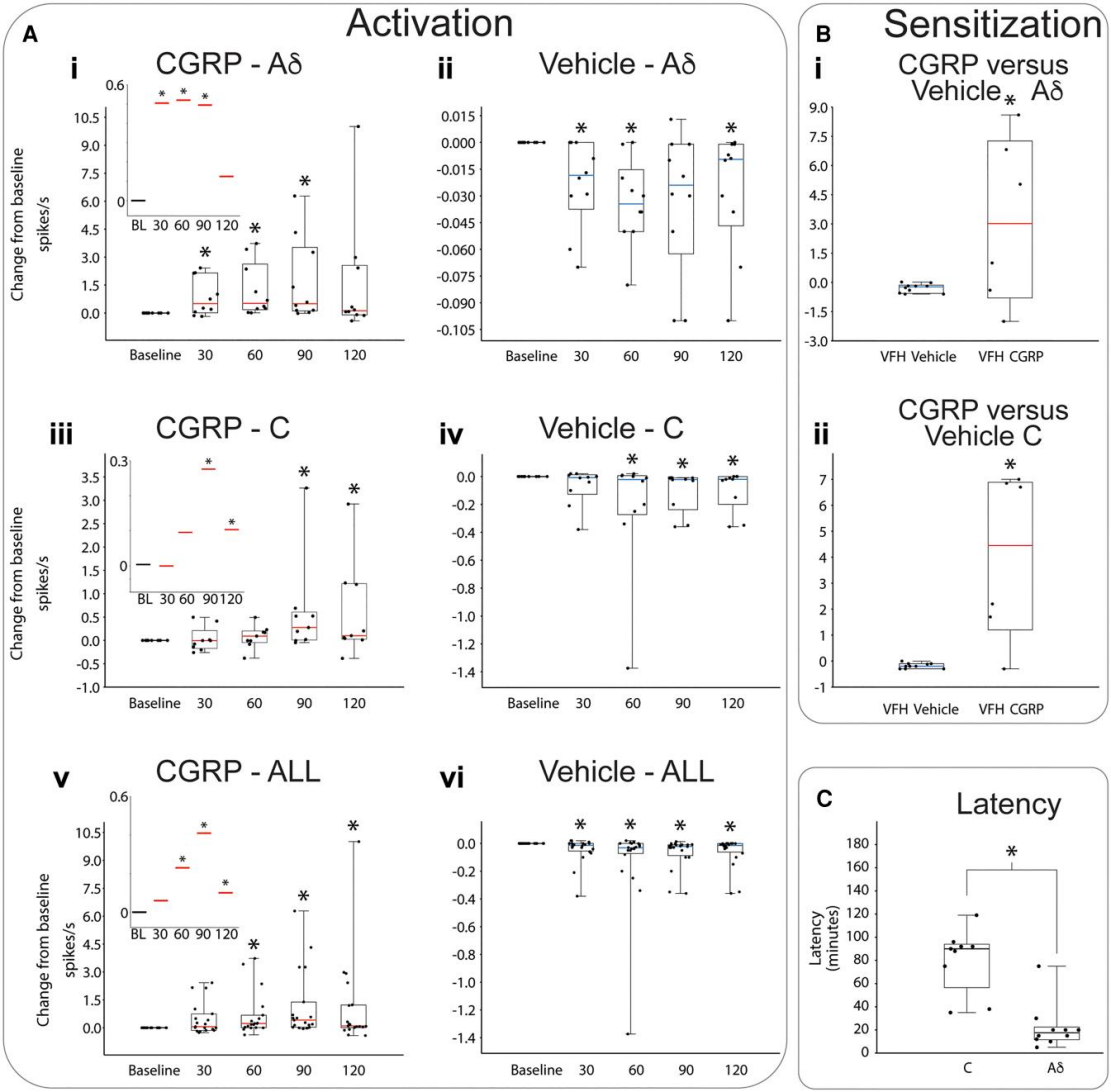
**Quantitation of activation, sensitization and response latency of meningeal Aδ- and C-fibres**. [**A**(**i**–**vi**)] Changes from baseline in ongoing firing averaged in 30-min intervals following CGRP or saline infusion, in Aδ, C-fibres and both samples combined (All). *P < 0.05 Friedman test; *post hoc*/Tukey's HSD. [**B** and **C**] Change from baseline in response to dural stimulation (von Frey hair) 2 h following infusion of CGRP or saline, in Aδ- [**B**(**i**)] and C-fibres [**B**(**ii**)], and latency of activation following CGRP infusion (**C**). *P < 0.05 Mann–Whitney U-test. Note that Aδ-fibres have a significantly shorter latency after CGRP infusion than C-fibres. *n*: **A**(**i**, **ii** and **iv**), *n* = 10; **A**(**iii**), *n* = 9; **A**(**v**), *n* = 19; **A**(**vi**), *n* = 20; **B**(**i** and **ii**), *n* = 6 Aδ, *n* = 6; C (CGRP) and *n* = 10 Aδ, *n* = 10 C (saline); **C**  *n* = 10 Aδ, *n* = 9 C. Box and whisker plots depict median and interquartile range, with scatter plots of values for individual neurons. For greater clarity, *insets* in **A**(**i**, **iii** and **v**) show plots of median values alone on an expanded scale. CGRP = calcitonin gene-related peptide; VFH = von Frey hair; Tukey's HSD = Tukey's honestly significant difference.

**Figure 3:**
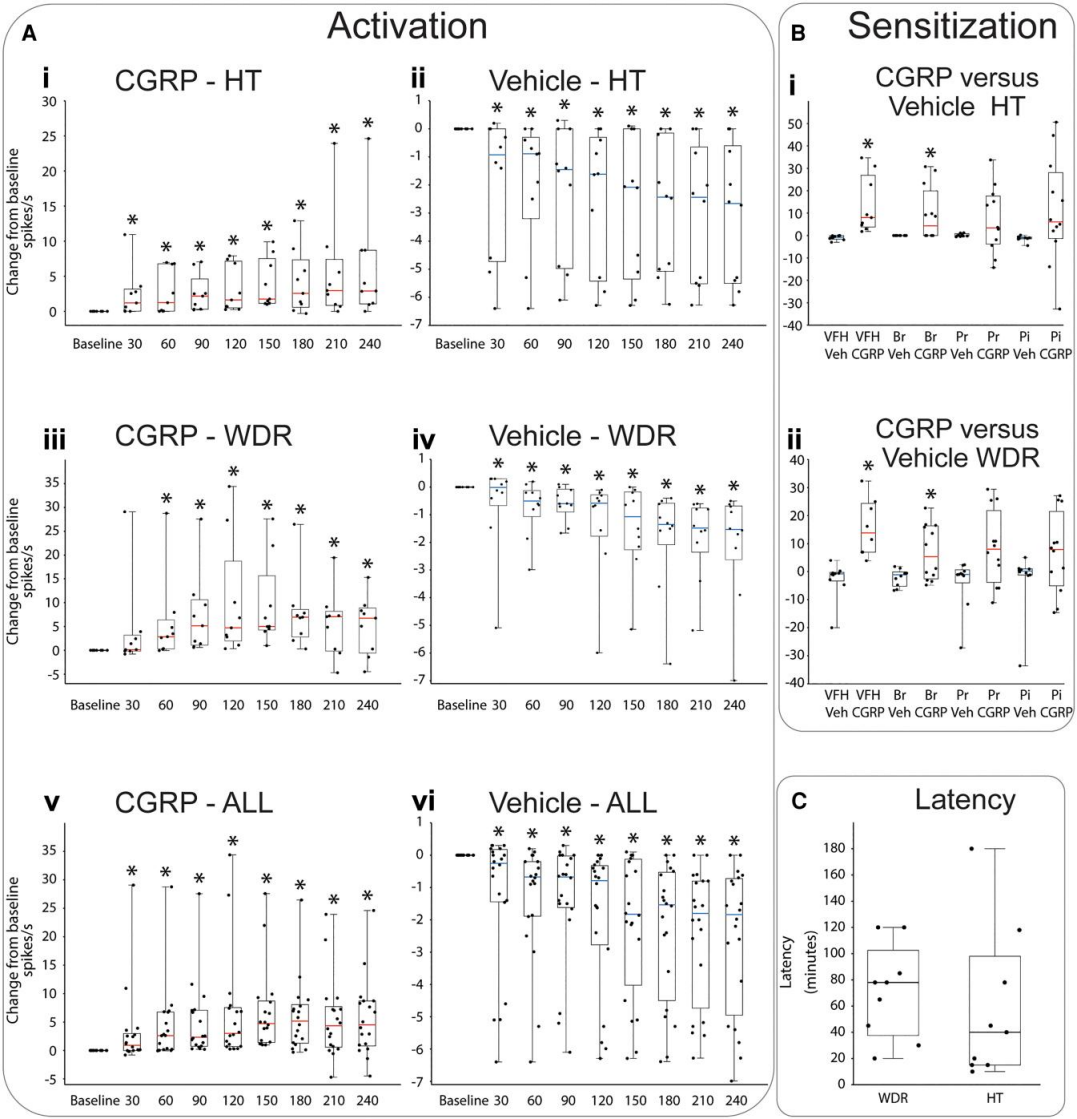
**Quantitation of activation, sensitization and response latency of HT and WDR dura-sensitive neurons.** [**A**(**i**–**vi**)] Changes from baseline in ongoing firing averaged in 30-min intervals following CGRP or saline infusion, in high threshold (HT), wide dynamic range (WDR) and both samples combined (All). *P < 0.05 Friedman test; *post hoc*/Tukey's HSD. [**B**(**i** and **ii**) and **C**] Change from baseline in response to dural (von Frey hair, VFH) and facial stimulation [brush (Br), pressure (Pr), pinch [Pi)] 2 h following infusion of CGRP or saline, in HT [**B**(**i**)] and WDR [**B**(**ii**)], and latency of activation following CGRP infusion (**C**). *P < 0.05 Mann–Whitney U-test. *n*: **A**(**i** and **iii**), *n* = 9; **A**(**v**), *n* = 18; **A**(**ii** and **iv**), *n* = 10; **A**(**vi**), *n* = 20; **B**(**i** and **ii**), *n* = 9 (CGRP), *n* = 10 (saline); **C**, *n* = 9 HT, *n* = 9 WDR. Box and whisker plots depict median and interquartile range, with scatter plots of values for individual neurons. CGRP = calcitonin gene-related peptide; Tukey's HSD = Tukey's honestly significant difference.

However, when delivered by intra-carotid infusion at the human dose (5 µg/kg/min for 20 min), CGRP, but not saline vehicle, produced sustained activation of first- and second-order neurons, as well as sensitization of their dural and facial receptive fields. Intra-carotid CGRP infusion produced increased firing in 19/32 first-order neurons (59%; 10/16 Aδ, 9/16 C-fibres) and 18/24 second-order dorsal horn neurons (75%; 9/12 HT, 9/12 WDR) (Figs 1–3). In control experiments, intra-caroid infusion of saline vehicle produced no activation in first-order or second-order neurons (*n* = 10 Aδ, 10 C-fibres, 10 HT, 10 WDR) (CGRP versus saline, *P* < 0.005 for Aδ, C-fibres; *P* < 0.001 for HT, WDR, Fisher's exact; Table 1 and Figs 1–3).

**Table 1:** Statistical comparisons

Neuron type	Condition or parameter	Comparison median [IQR] (*n*)	Statistic	*P*-value	Test
Aδ	Baseline firing	CGRP versus saline 0.4 [0.07–0.6] versus 0.3 [0.06–1.1] (16,10)	Z = 0.02	0.97	Mann–Whitney
C-fibre	Baseline firing	CGRP versus saline 0.5 [0.2–1.06] versus 0.5 [0.2–1.01] (16,10)	Z = 0.03	0.97	Mann–Whitney
Aδ	Post-infusion activation	CGRP versus saline incidence of activation (10/16 versus 0/10)	χ^2^ = 10.1	0.0025	Fisher's exact
C-fibre	Post-infusion activation	CGRP versus saline incidence of activation (9/16 versus 0/10)	χ^2^ = 8.6	0.0038	Fisher's exact
Aδ	CGRP dural response	Dural response. Change from baseline 3.02 [−0.8–7.3] (6)	Z = 1.4	0.026	Wilcoxon
Aδ	Saline dural response	Dural response. Change from baseline −0.2 [−0.6 to −0.1] (10)	Z = 0.9	0.99	Wilcoxon
C-fibre	CGRP dural response	Dural response. Change from baseline 4.5 [1.2–6.9] (6)	Z = 1.9	0.046	Wilcoxon
C-fibre	Saline dural response	Dural response. Change from baseline −0.2 [−0.3 to −0.1] (10)	Z = 1.1	0.88	Wilcoxon
Aδ versus C	Latency	Ad versus C: 17.5 [11.5–22.5] versus 90 [56.5–94] (10,9)	Z = 3.4	0.0005	Mann–Whitney
HT	Baseline firing	CGRP versus saline 1.28 [0.02–3.6] versus 6 [1.9–8.7] (12,10)	Z = 1.5	0.13	Mann–Whitney
WDR	Baseline firing	CGRP versus saline 4.7 [0.6–11.5] versus 5.9 [2.2–10.3] (12,10)	Z = 0.7	0.48	Mann–Whitney
HT	Post-infusion activation	CGRP versus saline incidence of activation (9/12 versus 0/10)	χ^2^ = 12.7	0.0004	Fisher's exact
WDR	Post-infusion activation	CGRP versus saline incidence of activation (9/12 versus 0/10)	χ^2^ = 12.7	0.0004	Fisher's exact
HT	CGRP dural response	Change from baseline 8.0 [3.8–26.9] (12)	Z = 2.7	0.003	Wilcoxon
HT	Saline dural response	Dural response. Change from baseline −0.6 [−1.5 to −0.1] (10)	Z = 0.5	0.59	Wilcoxon
WDR	CGRP dural response	Change from baseline 13.9 [7–24.4] (12)	Z = 2.5	0.007	Wilcoxon
WDR	Saline dural response	Change from baseline −0.9 [−3.4 to −0.3] (10)	Z = 1.0	0.3	Wilcoxon
HT	CGRP skin brush	Change from baseline 4.3 [0–19.9] (12)	Z = 2.2	0.031	Wilcoxon
WDR	CGRP skin brush	Change from baseline 13.6 [7.0–18.6] (10)	Z = 1.9	0.044	Wilcoxon
HT versus WDR	Latency	HT versus WDR: 40 [15–98] versus 78 [37.5–102.3] (9,9)	Z = 0.53	0.59	Wilcoxon
Central	CGRP injection into ganglion	Firing rate increase (9)	χ^2^ = 12.5	0.0008	Friedman
		1 h versus baseline		0.042	Tukey's HSD
		2 h versus baseline	–	0.011	Tukey's HSD
HT + WDR	CGRP activation rate	Male versus female	–	1	Fisher's exact
HT + WDR	Post-infusion activity	Male versus female	–	0.98	Mann–Whitney
WDR	CGRP infusion	Male versus female incidence of activation (3/4 versus 6/8)	–	1	Fisher's exact
WDR	Male versus female	Baseline firing male versus female	–	0.93	Mann–Whitney
HT	Male versus female	CGRP activation (2/3 versus 7/9)	–	1	Fisher's exact
HT	Male versus female	Baseline firing	–	0.28	Mann–Whitney
C-fibre	Male versus female	CGRP activation (3/5 versus 6/11)	–	1	Fisher's exact
C-fibre	Male versus female	Baseline firing	–	0.66	Mann–Whitney
Aδ-fibre	Male versus female	CGRP activation (3/5 versus 6/11)	–	1	Fisher's exact
Aδ-fibre	Male versus female	Baseline firing	–	0.82	Mann–Whitney

CGRP = calcitonin gene-related peptide; HT = high threshold; Tukey's HSD = honestly significant difference; WDR = wide dynamic range.

There was a difference between the neuronal subgroups in the temporal pattern of post-CGRP activation, in that the increase in firing rate in the Aδ fibres and the HT neurons (which receive predominantly Aδ input)^23,24^ was significant in the first 30-min interval after the start of infusion [Figs 1A, C, 2A(i) and 3A(i)], whereas the C-fibres and the WDR neurons (which receive predominantly C-fibre input)^23,24^ showed a delay before the start of activation [significant increase above baseline not beginning until the second (HT neurons) or third (C-fibres) 30-min post-infusion interval] [Figs 1B, D, 2B(iii) and 3B(iii)]. In the control experiments, there was a tendency for firing to decrease over time relative to baseline, which is typical of unstimulated control neurons in our studies [Figs 2A(ii and iv) and 3(ii and iv)].

In addition to activation, intra-carotid CGRP also produced sensitization of both the first- and second-order neurons to mechanical stimulation of their peripheral receptive fields. Enhanced responses to dural indentation with a calibrated von Frey monofilament were found in 12/19 first-order neurons (63%; 6/10 Aδ, 6/9 C) and 17/24 second-order dorsal horn neurons (70%; 9/12 HT, 8/12 WDR). Response amplitudes were significantly increased above baseline (*P* < 0.05 and *P* < 0.01 for first- and second-order neurons, respectively). In the control groups, no neurons showed sensitization to dural indentation (*n* = 20 each for first-order and second-order neurons) [Figs 2B(i and ii), 3B(i and ii) and Table 1). In addition to enhancement of dural responses, intra-carotid CGRP also enhanced facial responses of the second-order dorsal horn neurons. Responses to brush increased in both HT and WDR neurons (increase of 4.3 and 5.4 Hz, *P* = 0.03 and 0.04, respectively). Responses to pressure and pinch did not increase significantly. In the control groups, none of the neuronal populations showed a significant change in responses to peripheral stimulation [Fig. 3B(i and ii) and Table 1].

### Non-dural neurons

**Figure 4:**
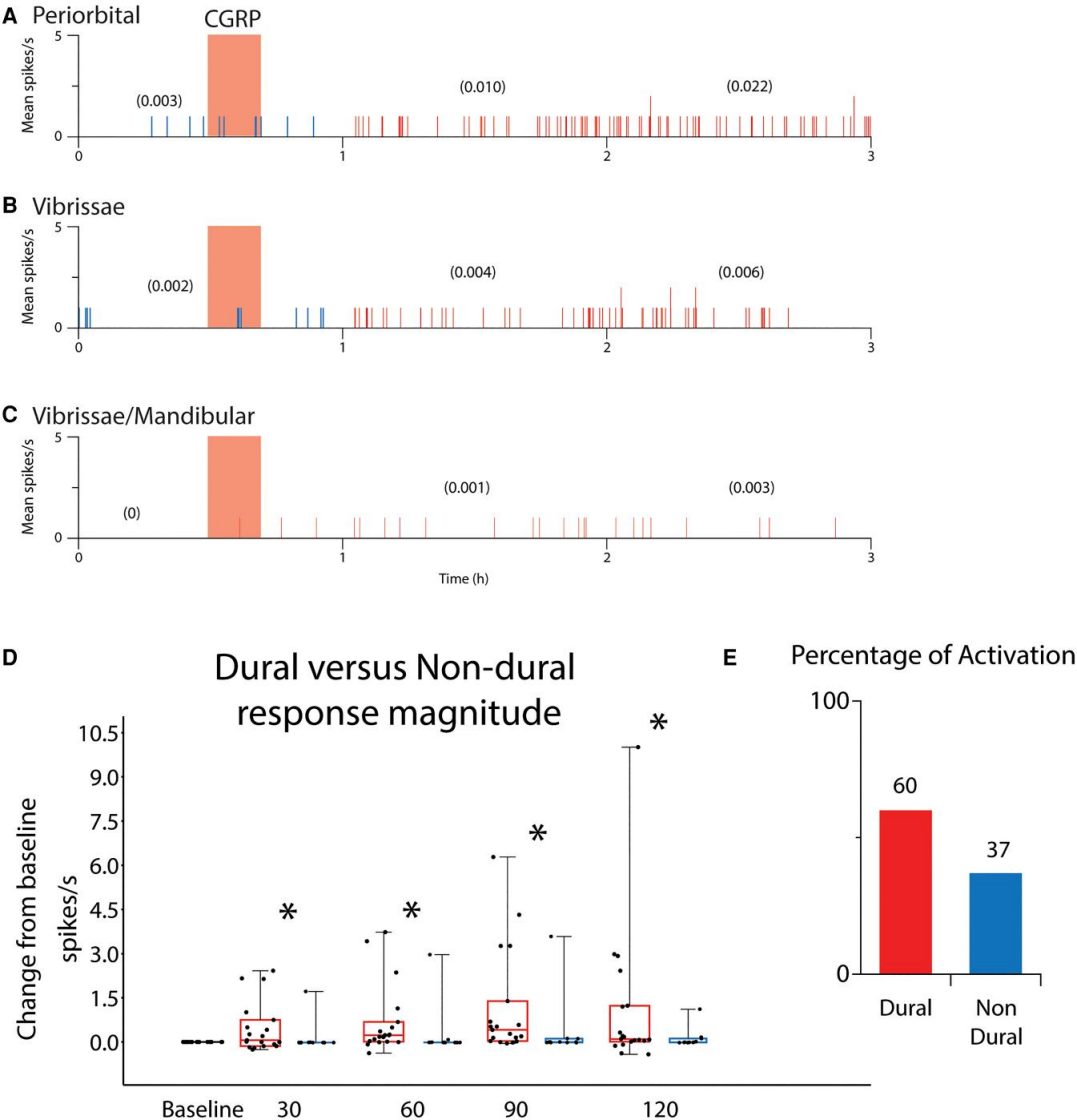
**Intra-carotid CGRP infusion activates non-dural neurons with skin receptive fields**. Plots of firing rate in examples of individual neurons with skin receptive field in the periorbital, vibrissae and vibrissae/mandibular regions tested for activation by intra-carotid infusion of CGRP (50 ng/kg/min for 20 min) (**A**–**C**). Activity plotted in red indicates significant increase above baseline (1 standard deviation). (**D**) Changes from baseline in ongoing firing averaged in 30-min intervals following CGRP infusion of dural versus non-dural neurons. (**E**) Percentage of activation of dural and non-dural neurons. *P < 0.05 Mann–Whitney U-test. CGRP = calcitonin gene-related peptide.

For comparison with the dural primary afferent neurons, we recorded from a sample of primary afferent neurons in the trigeminal ganglion with facial but not dural receptive fields to test their responses to intra-carotid CGRP. Previous anatomical studies have documented that individual trigeminal ganglion neurons innervate either intracranial or extracranial tissues, but not both, in the adult rats,^2,25^ so neurons with dural and facial receptive fields are separate, non-overlapping populations. The non-dural neurons showed a significantly lower incidence of activation in response to the CGRP infusion than the dural neurons (11/30, 37% versus 60% for the dural neurons, *P* = 0.04, Fisher's exact); activation was found in 5/19 neurons with receptive fields in the peri-orbital region and 6/11 with receptive fields in the mandibular, vibrissal or snout regions. Notably, the activation observed in these neurons was generally weak: in 8 of the 11 activated non-dural neurons, the total spike count over the 2-h post-infusion recording period was fewer than 100 spikes. The overall magnitude of the response was significantly smaller than in the dural neurons [non-dural: 0.007 (0.002–0.021) *n* = 11; dural: 0.19 (0.002–0.945) *n* = 19, median (interquartile range)] (Mann–Whitney U-test, *P* < 0.05) (Fig. 4).

### Peripheral anaesthethic blockade

**Figure 5:**
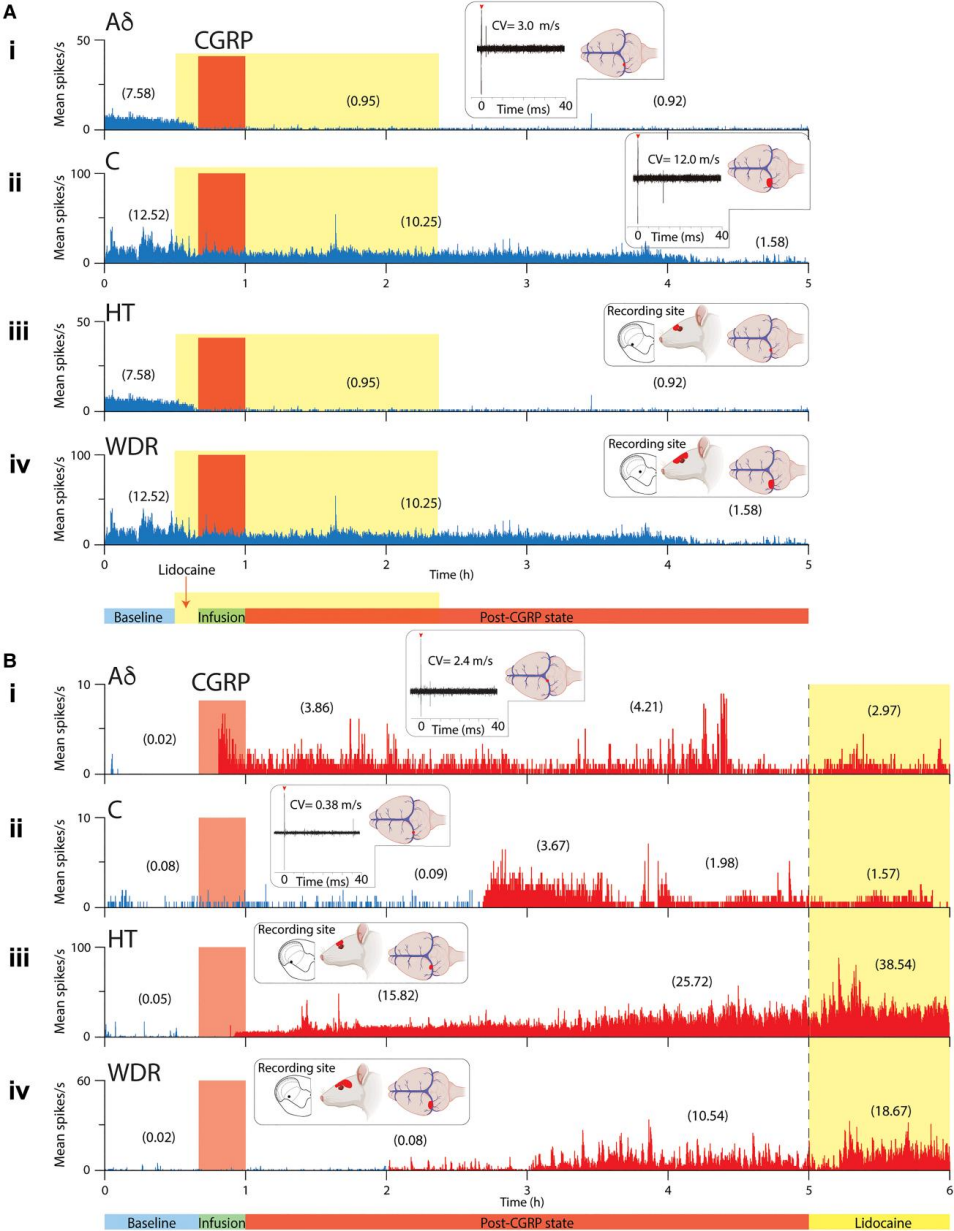
**Activation of meningeal nociceptors and second-order dura-sensitive neurons by intra-carotid infusion of CGRP could be prevented but not reversed by administration of lidocaine to the dura**. Individual examples of experiments in which local anaesthetic (lidocaine 2%, 10 ml) was applied to the dura either 10 min before (**A**) or 4 h after (**B**) intra-carotid infusion of CGRP, in Aδ, C, high threshold (HT) and wide dynamic range (WDR) neurons. No activation was observed by CGRP after numbing of the dura with lidocaine (**A**); there was no significant change from baseline in any 30-min interval following the start of CGRP infusion for any of the four neuronal subpopulations (median change from baseline in each 30-min interval ranged from −0.3 to 0.02 for the first-order neurons, and −0.7 to −0.01 for the second-order neurons). Delayed application after activation had been induced by CGRP infusion was not able to reverse the activation (**B**). Other details as in Fig. 1. CGRP = calcitonin gene-related peptide. Figure partially created in BioRender. Melo Carrillo, A. (2026) https://BioRender.com/n1j7o1s.

To investigate the possible site of action of CGRP in producing the nociceptive neuronal activation, experiments were done in which lidocaine was applied to the dura 15 min before the start of the CGRP infusion, and continuing for 2 h, at which time the dura was washed with saline to remove the lidocaine. In the presence of dural lidocaine, CGRP failed to produce activation or sensitization in any of the first- or second-order neurons tested (0/6 and 0/6, respectively; significant difference compared with experiments without lidocaine, *P* < 0.0005, Fisher's exact, Fig. 5A). This finding indicates that the activation of both the first- and second-order neurons by intra-carotid CGRP infusion depends on its action in the dura, presumably through activation of the dural nerve endings of meningeal primary afferent nociceptors. Following removal of the lidocaine, in the second hour post-infusion, no increase in ongoing firing or neuronal sensitization was observed in any of the neurons, which suggests that the effects of CGRP that result in the neuronal activation occur primarily within the first hour post-infusion.

To investigate this observation, we tested an additional set of neurons using a modified experimental paradigm, in which the lidocaine was applied 2 h after the neuronal activation had become evident; only neurons that showed a sustained activation following the CGRP infusion were tested in this paradigm. Application of lidocaine post-CGRP did not alter the increased firing activity or enhanced receptive field responses of any of the neurons tested in this paradigm (*n* = 4 first-order and four second-order neurons, Fig. 5B). These results suggest that: (i) the second-order neurons may sustain their increased firing independent of peripheral input once activation persists beyond 1 h; and (ii) the activity of the first-order neurons, once sensitized, may be maintained intrinsically at the level of the soma, rather than being continually driven by activation of their peripheral nerve endings in the dura.

Given these findings and the observed effects of peripheral dural input blockade, we next investigated whether similar outcomes could be achieved in the second-order neurons by blocking the trigeminal ganglion directly with injection of local anaesthetic into the ganglion prior to the CGRP infusion, thereby completely interrupting peripheral input to second-order neurons (*n* = 4). Anaesthetic blockade of the ganglion abolished both the ongoing discharge of the second-order neurons as well as their responses to dural and facial stimulation. None of the neurons tested in this paradigm showed activation or sensitization of their peripheral receptive fields following intra-carotid CGRP infusion (*n* = 4). In addition, the ongoing discharge of the neurons dropped to zero following the lidocaine application. Thus, the ganglionic blockade was effective in eliminating both CGRP-mediated activation as well as baseline activity driven from ongoing peripheral input, whether dural or non-dural.

**Figure 6:**
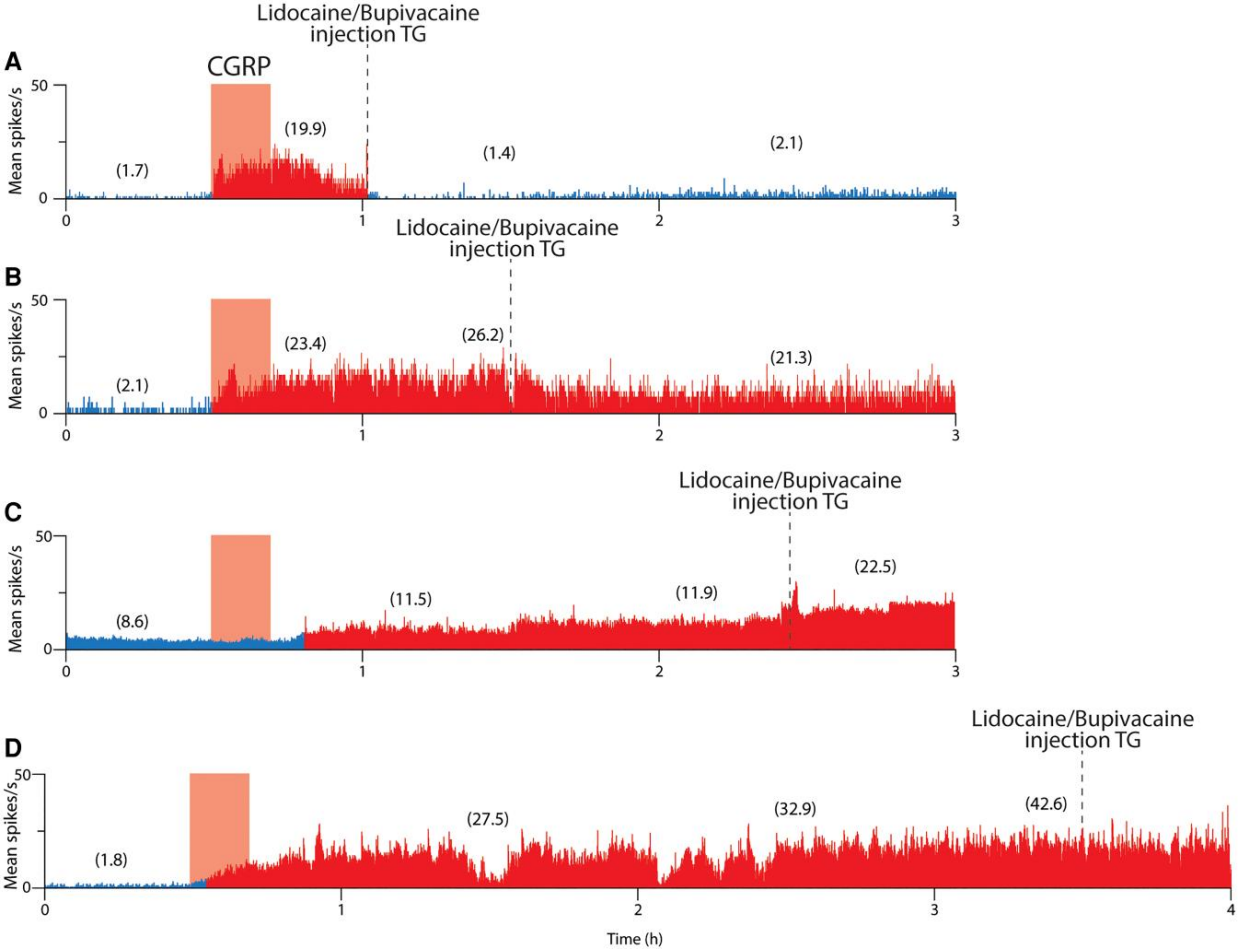
**Activation of second-order dura-sensitive neurons by intra-carotid infusion of CGRP could be attenuated by early but not late injection of lidocaine/bupivacaine into the trigeminal ganglion**. Examples of individual experiments in which local anaesthetic (10% lidocaine/10% bupivacaine, 1% Chicago Blue, 10 μl total) was injected into the trigeminal ganglion (TG) at different time points after intra-carotid CGRP infusion. The anaesthetic greatly reduced the CGRP-induced activation when injected at 30 min (**A**), showing that the CGRP-induced increment in ongoing activity was driven by the periphery. In an experiment in which the ganglion injection was made at a later time point (1, 2 and 3 h after the start of CGRP infusion), the neuron’s activity was maintained and actually increased, showing that the activity had become independent of peripheral input and was driven by central changes. CGRP = calcitonin gene related peptide.

However, a key question remained: at what time point following CGRP infusion does activation and sensitization of central neurons become independent of peripheral input? To address this, we performed trigeminal ganglion blockade at varying time points after the start of the CGRP-induced activation—specifically, 30 min, 1 , 2 and 3 h after the onset of neuronal activation. These experiments were conducted on four second-order dura-sensitive neurons. As shown in Fig. 6, only when the blockade was applied within the first hour after activation was the CGRP-induced activation successfully inhibited. These results indicate that the activation and central sensitization are dependent on peripheral actions of CGRP occurring within the first hour after infusion, and after 1 h become independent of continued peripheral input. Importantly, this sensitization can be prevented if peripheral input is blocked within the first 30 min. Taken together, these findings reinforce previous evidence suggesting that early intervention—in this case, within 30 min of CGRP infusion—is critical to prevent activation of the meningeal sensory pathway.

### Ganglionic injections

**Figure 7:**
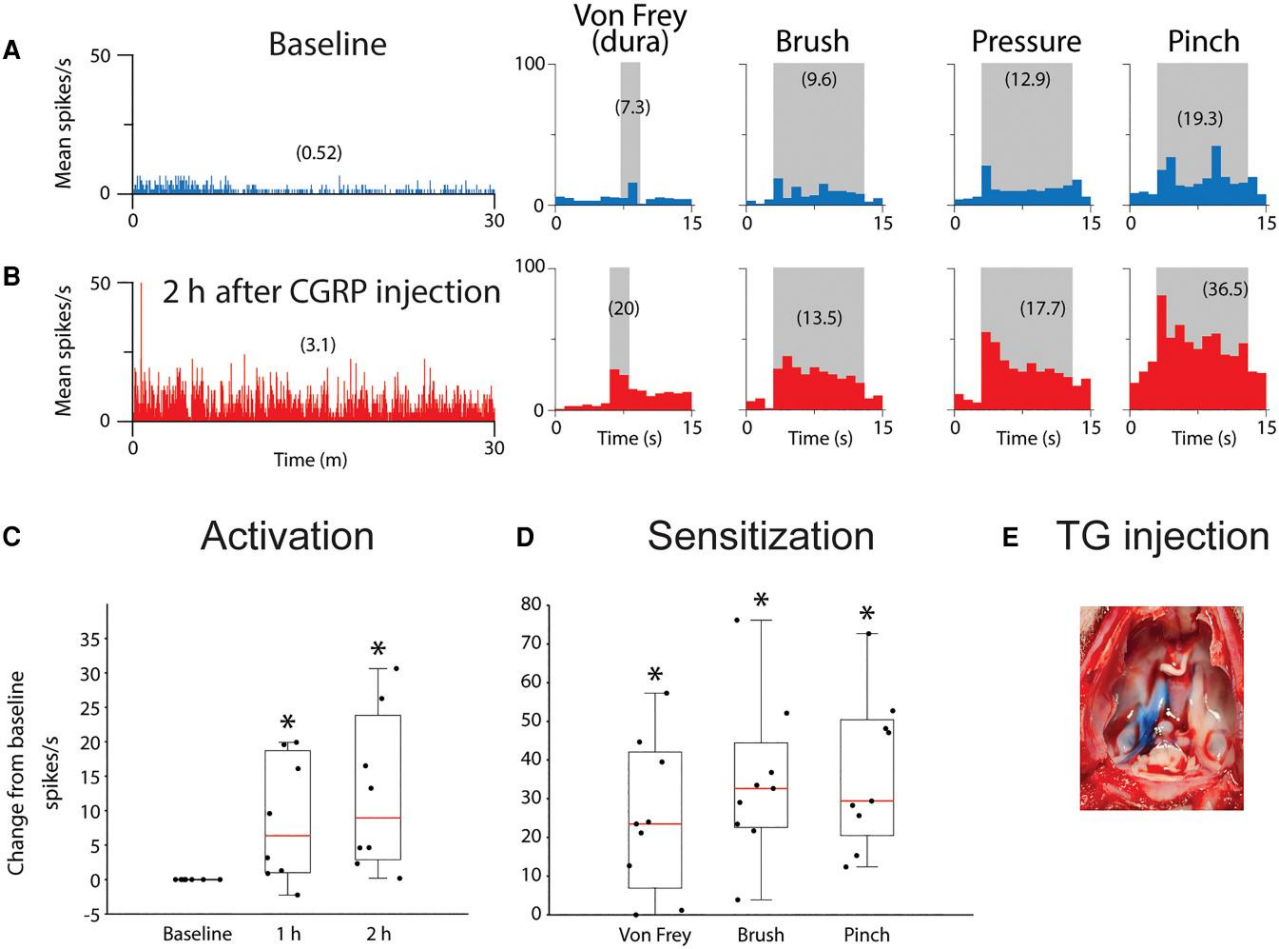
**CGRP injection into the trigeminal ganglion activates and sensitizes dura-sensitive HT and WDR neurons.** (**A** and **B**) Example of activity in an individual second-order (WDR) neuron showing ongoing firing and responses to dural (von Frey) and facial stimulation (brush, pressure, pinch) during baseline (**A**) and 2 h after injection (300 ng CGRP, 10 ml) (**B**). (**C** and **D**) Change from baseline in ongoing firing during first and second hour post-injection (**C**) and in responses evoked from dural (von Frey) and facial stimulation (brush, pinch) at 2 h following injection (**D**), in a sample of nine second-order dural-responsive neurons. *P < 0.05 Friedman test; *post hoc*/Tukey's HSD (**C**); *P < 0.05 Wilcoxon signed rank test (**D**). Box plots (median, interquartile range) with scatter plots of values for individual neurons. (**E**) Post-mortem photograph to document the degree of filling of the trigeminal ganglion (TG). CGRP = calcitonin gene-related peptide; HT = high threshold; Tukey's HSD = honestly significant difference; WDR = wide dynamic range.

To investigate the potential sites of action of CGRP in producing the central neuronal activation, CGRP was injected into the trigeminal ganglion, and activity of second-order neurons was recorded for 2 h after injection (*n* = 5 HT and 4 WDR neurons). Ganglionic injection of CGRP produced activation in 8/9 second-order neurons (88%; Fig. 7  Table 1). Discharge was increased significantly above baseline during the first and second hour following the injection (Table 1). All nine neurons were sensitized to dural and facial stimulation.

### Male versus female

Data collected pre- and post-CGRP infusion revealed no differences between first- or second-order neurons recorded in male and neurons recorded in female rats, regardless of whether they were Aδ, C, HT or WDR (Table 1). There were no significant differences between males and females in baseline rates or in the magnitude of the post-CGRP infusion increases in activity. We therefore combined all data collected in males and females in the overall analyses.

## Discussion

Using a novel method for studying CGRP role in migraine, we show that low-dose intra-carotid CGRP infusion produced sustained activation and sensitization of first- and second-order neurons of the meningeal sensory pathway. Similar effects were produced by CGRP injection into the trigeminal ganglion. These CGRP effects were impeded by local anaesthetic blockade of the dura or trigeminal ganglion before but not 1 h after CGRP infusion. These findings provide the first evidence of peripheral nociceptive neuronal activation by CGRP, identify the dura mater as a critical site of CGRP action in initiating migraine-related nociceptive signalling, support a rationale for early administration of peripherally acting CGRP-targeted migraine treatments and call for continuous effort to focus further development of anti-migraine drugs that act peripherally rather than centrally as such drugs are far less likely to introduce unwanted side effects.

Similar to previous findings,^20^ we also found that intravenous infusion of CGRP at the human dose failed to produce nociceptive neuronal activation. The idea behind trying the intra-carotid route was to produce a regional infusion for more effectively targeting the cranial tissues, especially the cranial meninges, before it has a chance to undergo greater dilution in the systemic circulation and exposure to possible degradation by the liver. This change in route allowed us to achieve nociceptive neuronal activation without resorting to raising the dose to non-physiological levels and potentially inducing effects unrelated to CGRP’s role in headache. The use of intra-carotid infusion, a systemic method of administration, raises the question of where the CGRP might be acting to produce the nociceptive neuronal effects. Our experiments with lidocaine addressed this question by showing that the neuronal effects are absent when the CGRP infusion is preceded by local anaesthetic blockade of either the dura or the trigeminal ganglion. This is evidence that CGRP’s nociceptive neuronal effects result from an action on sensory nerve terminals in the dura, which in turn leads to primary afferent activation and central transmission.

The sustained neuronal activation and sensitization induced by intra-carotid CGRP infusion were found to be dependent on an initial peripheral mechanism. This conclusion is supported by our finding that the application of lidocaine to either the dura mater or the trigeminal ganglion effectively abolished CGRP-induced neuronal responses when administered prior to or within 40 min of the infusion. These results suggest that CGRP initiates a cascade of events at the peripheral terminals of meningeal afferents which, if not interrupted during this early phase, culminate in sustained central sensitization. Notably, lidocaine application at later time points—particularly beyond 2 h post-infusion—was no longer effective in reversing neuronal activation, highlighting the existence of a defined temporal window during which peripheral input is necessary to establish and consolidate central sensitization. This temporal dependence aligns with clinical evidence indicating that early intervention is critical for the successful treatment of migraine attacks.^26-31^ The therapeutic efficacy of acute pharmacological agents has been shown to decline markedly once the attack progresses beyond the initial phases, likely corresponding to the transition from a peripherally driven to a centrally maintained state of sensitization. Our findings offer a mechanistic basis for this clinical observation and underscore the importance of early targeting of peripheral pathways—particularly CGRP-related signalling—before central mechanisms become independently self-sustaining.

We observed a clear temporal difference in activation between peripheral Aδ-fibre nociceptors and C-fibres: Aδ-fibres were activated significantly earlier. This latency may reflect the differential expression of CGRP receptors; only Aδ-fibres express the canonical CGRP receptor and are likely to respond directly and rapidly to CGRP, whereas C-fibre activation may arise secondarily, perhaps due to neurogenic inflammation or, in the cases where their activation occurred faster, due to direct action via amylin 1 receptors, which are also expressed on C-fibres.^32,33^ While these differences in latency were not completely preserved in the second-order neurons (possibly due to the fact that Aδ fibres terminate on both HT and WDR neurons), the finding points to early activation in the Aδ-HT pathway and delayed activation in the C-WDR pathway. Theoretically, it is reasonable to consider the possibility that the immediate and brief headache seen in the human CGRP provocation studies^9,10^ could be mediated by the CGRP effects on the Aδ-HT pathway whereas the delayed migraine-like headache could be mediated by the CGRP effects on the C-WDR pathway. Furthermore, it may be that the immediate headache requires activation of only one of these two pathways whereas the migraine-like headache requires recruitment (i.e. activation and sensitization) of both pathways.

One particularly intriguing aspect of CGRP biology is its apparent specificity for inducing cranial pain, despite its widespread expression throughout the peripheral nervous system.^1-3^ While this study was not designed to resolve this longstanding question, our data do provide evidence supporting the preferential activation of dura-sensitive neurons by CGRP. In recordings from trigeminal ganglion neurons lacking dural receptive fields—such as those innervating the vibrissal pad or cutaneous facial areas—the incidence and magnitude of CGRP-induced activation were significantly lower than in dura-sensitive neurons. Previous studies, including those by Gold and colleagues,^34-36^ have proposed that neuronal subpopulations may exhibit distinct CGRP receptor profiles or signal transduction pathways, which could account for their selective sensitivity. In this context, our findings raise the possibility that the heightened responsiveness of dura-innervating neurons to CGRP reflects an intrinsic specialization of this afferent population—potentially a key factor underlying the cranioselectivity of migraine pain. Regarding CGRP ability to provoke headache but not any other pain, we also note that in most cases in which non-dura sensitive neurons became activated by the CGRP infusion, their activation amounted to a total of less than 100 spikes over a period of 2 h (i.e. about 0.01 spikes/s), likely to be far below the activation rate required to produce the perception of pain.

One of the more surprising findings of our study is the activation of central trigeminovascular neurons by intraganglionic infusion of CGRP into the trigeminal ganglion. It is surprising because peripheral sensory neurons use receptors in their synaptic terminals to detect stimulants and send signals through their cell somas to the dorsal horn. Activation that originates in cell bodies provide a vastly untraditional way for flow of sensory signals into the CNS. Even more surprising is the finding that local administration of lidocaine to the dura blocks activation of trigeminovascular neurons by intra-carotid administration of CGRP, as this observation questions the significance of intraganglionic CGRP activation of meningeal nociceptors. One possible explanation for this apparent conflicting finding is that the amount of CGRP that reaches the ganglion following systemic administration is too low to trigger neuronal activation. Translating into humans, it may be that the amount of CGRP released during migraine is high enough in the dura to activate the nociceptors from their peripheral terminals but is not sufficient to activate the cell somas in the ganglion.

Recently, we showed that PACAP (pituitary adenylate-cyclase-activating polypeptide) is capable of activating and sensitizing both C- and A-δ meningeal nociceptors.^37^ These findings suggest that CGRP is not the only neuropeptide capable of activating the trigeminovascular pathway at its origin in the meninges. Similar to CGRP, PACAP is also capable of inducing mild headache in healthy controls and migraine attacks in individuals with migraine.^38^ Additionally, both peptides are capable of dilating cerebral blood vessels^7,39-41^ and inducing migraine-like symptoms in mouse models of intracranial nociception, including mechanical allodynia and photophobia.^42-45^ It is equally important to note differences that may have implications to migraine pathophysiology and treatment outcomes. As shown recently, intravenous CGRP induces dilatation of dural but not pial arteries, while agents that block CGRP action prevent CGRP-evoked dilatation of dural arteries but not dilatation of dural or pial arteries evoked by cortical spreading depression.^7^ Theoretically, it is reasonable to suggest that other neuropetides may be involved in these dilatations. Given the presence of PACAP (but not CGRP) in parasympathetic postganglionic neurons,^46^ it is possible that the roles these two peptides play in migraine pathophysiology differ. Supporting this view are data showing that CGRP and PACAP are expressed in largely separate populations of trigeminal ganglion neurons.^47^

Finally, we observed no significant differences between male and female animals in baseline neuronal activity or in responses induced by CGRP infusion, suggesting that the fundamental mechanisms of peripheral activation and central sensitization within the trigeminovascular pathway are largely conserved across sexes. This absence of sex differences in electrophysiological recordings at the level of the brainstem and trigeminal ganglion implies that the increased incidence and severity of migraine reported in females^48-50^ are unlikely to originate from heightened excitability or sensitization at the level of the peripheral or early central nociceptive neurons. Instead, our findings support the hypothesis that sex-related differences in migraine pathophysiology are more likely to be mediated by downstream brain regions responsible for the integration and modulation of pain signals, such as the thalamus, insula and prefrontal cortex, which are known to exhibit sex-specific connectivity and functional activation patterns. These areas may amplify or differentially interpret the nociceptive inputs initiated by CGRP, contributing to the behavioural disparities observed between sexes. Thus, while CGRP may activate the trigeminovascular system in a sex-independent manner, its effects on migraine expression and symptomatology could still vary significantly due to central, supraspinal mechanisms. It is also important to note that as of now, there are no sufficiently powered clinical trials to support the claim that CGRP inhibitors are only effective in females and not in males.^51^ In the absence of strong evidence (e.g. randomized controlled studies where males and females are represented equally), clinicians and scientists must be careful in assuming that the sex differences found in animal models should be used to recommend preferential treatment for migraine in women over men.

## Conclusions

Intra-carotid—but not systemic—administration of CGRP activates and sensitizes first- and second-order neurons of the meningeal sensory pathway in rats. These effects are mediated through peripheral sites of action—most notably the dura—and rely on peripheral input within the first 40–60 min following infusion. Blocking this input within that window prevents central sensitization whereas delayed blockade is ineffective. These results underscore the pivotal role of peripheral CGRP signalling in initiating migraine-related nociception and support the therapeutic rationale for early treatment of acute migraine attacks with CGRP-targeted therapies. Furthermore, by demonstrating a causal and spatially constrained role for CGRP in initiating trigeminovascular activation, our study offers a mechanistic explanation for the clinical efficacy of peripherally acting CGRP antagonists and monoclonal antibodies, and thus advances our understanding of the early events that trigger migraine pain.

## Data Availability

The data that support the findings of this study are available from the corresponding author, upon reasonable request.
